# Circulating argonaute-bound microRNA-126 reports vascular dysfunction and treatment response in acute and chronic kidney disease

**DOI:** 10.1016/j.isci.2020.101937

**Published:** 2020-12-13

**Authors:** Kathleen M. Scullion, A. D. Bastiaan Vliegenthart, Laura Rivoli, Wilna Oosthuyzen, Tariq E. Farrah, Alicja Czopek, David J. Webb, Robert W. Hunter, Matthew A. Bailey, Neeraj Dhaun, James W. Dear

**Affiliations:** 1 University/British Heart Foundation Centre of Research Excellence, Centre for Cardiovascular Science, University of Edinburgh, Queen's Medical Research Institute, 47 Little France Crescent, Edinburgh EH16 4TJ, UK

**Keywords:** Molecular Physiology, Molecular Genetics, Molecular Biology, Immunology

## Abstract

Vascular and kidney dysfunction commonly co-exist. There is a need for biomarkers of vascular health. Circulating microRNAs are biomarkers; miR-126 is endothelial cell-enriched. We measured circulating miR-126 in rats with nephrotoxic nephritis (NTN) and humans with acute endothelial and renal injury (vasculitis associated with autoantibodies to neutrophil cytoplasm antigens (ANCAs)). We compared these findings to those from patients with chronic kidney disease (CKD) and end-stage renal disease (ESRD) and explored the relationship between miR-126 and vascular dysfunction. In NTN, miR-126 was reduced. In ANCA vasculitis (N = 70), pre-treatment miR-126 was reduced compared to health (N = 60) (88-fold). miR-126 increased 3.4-fold post-treatment but remained lower than in health (∼26-fold). Argonaute 2-bound miR-126 increased with ANCA vasculitis treatment. miR-126 did not differ between CKD (N = 30) and health but its concentration correlated with endothelial dysfunction. miR-126 was reduced in ESRD (N = 15) (∼350 fold). miR-126 may be a marker of vascular inflammation and could aid decision-making.

## Highlights

• New biomarkers are needed that report vascular function in patients with kidney disease
• The endothelial microRNA, miR-126, was reduced with vascular inflammation (vasculitis)
• miR-126 correlated with vascular health in chronic kidney disease
• Circulating miR-126 may be a marker of vascular dysfunction

## Graphical abstract

**Figure:**
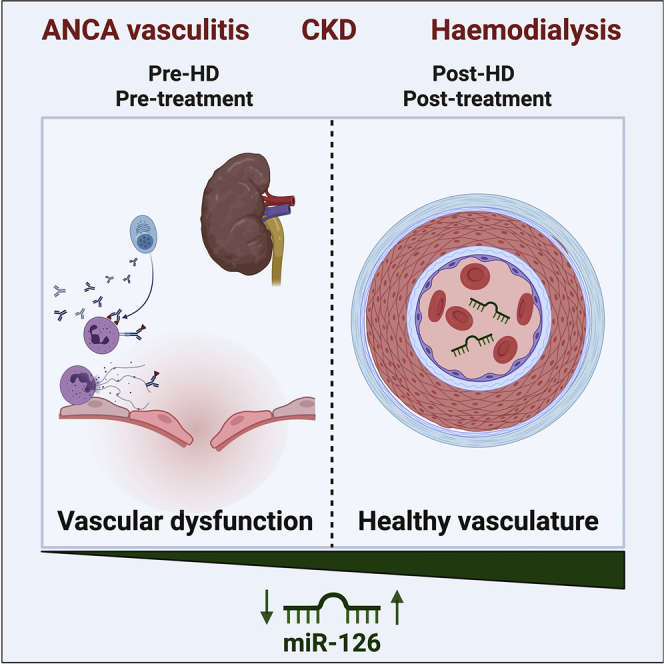

## Introduction

Vascular dysfunction commonly co-exists with kidney disease and contributes to an increased risk of cardiovascular disease (CVD). A severe, acute form of this vascular-renal phenotype is seen in patients presenting with anti-neutrophil cytoplasm antibody-associated vasculitis (AAV), a rare autoimmune disorder. The most frequent severe manifestations of AAV involve endothelial injury giving rise to a rapidly progressive glomerulonephritis and pulmonary hemorrhage. Despite current treatments overall survival remains poor (Flossmann et al., 2011; Hoffman et al., 1992) with many patients suffering chronic inflammation, a major contributor to the development and progression of both CVD (Hahn, 2003) and chronic kidney disease (CKD) (Landray et al., 2004). Furthermore, those who respond to treatment remain at risk of further disease relapses (Boomsma et al., 2000).

Identifying AAV early and assessing its response to treatment remain important clinical challenges. In those with renal involvement, the measurement of renal function using serum creatinine is often inadequate because substantial renal damage can occur before function is impaired to a detectable extent (Hewitt et al., 2004). Additionally, although serum creatinine may fall with treatment, it remains unclear whether histological inflammation continues once renal function has stabilized. Also, currently, there are no good measures of disease activity in those with extra-renal AAV alone. Biomarkers specific to small vessel inflammation would not only allow early implementation of appropriate treatments but also help identify those patients with grumbling disease activity and potentially predict disease relapses (Kitching et al., 2020).

While AAV might be considered a phenotypic extreme, patients with CKD due to any cause are at an increased risk of CVD (Foley et al., 1998). Indeed, those with CKD have a substantially higher chance of dying from CVD than of progressing to end-stage renal disease (ESRD) (Sarnak et al., 2003). Increased arterial stiffness, a marker of CVD risk (Blacher et al., 1999; Guerin et al., 2008), is a commonly recognized feature of CKD (Guerin et al., 2008), and an independent predictor of mortality and survival in these patients (Blacher et al., 1999; Guerin et al., 2001). The endothelium is an important regulator of arterial stiffness (Wilkinson et al., 2004), and endothelial dysfunction is also a common feature of CKD (Endemann and Schiffrin, 2004; van Guldener et al., 1997, 1998) and a predictor of CVD (Perticone et al., 2001).

MicroRNAs (miRs) are small (∼22 nucleotide-long) non-protein coding RNA species involved in post-transcriptional gene product regulation (Bartel, 2009). In blood, miRs are reported to be stable being protected from degradation by extra-cellular vesicles (such as exosomes), RNA binding protein complexes (such as argonaute 2 – Ago2) and lipoproteins (Mitchell et al., 2008; Zhou et al., 2013). As miRs are amplifiable and some are tissue restricted, they represent a new reservoir for biomarker discovery. For example, miR-122 is an established biomarker for liver injury (Starkey Lewis et al., 2011).

miR-126 is enriched in endothelial cells and is a regulator of vascular integrity and angiogenesis. The -3p and -5p forms of miR-126 have activity in endothelial cells – 3p is anti-inflammatory (Harris et al., 2008) and 5p is pro-proliferative (Schober et al., 2014). miR-126 is reported to be released from endothelial cells bound to Ago2 and in extra-cellular vesicles, which can transfer functional miR-126 into recipient cells to promote vascular repair (Zhou et al., 2013). When endothelial cells were stimulated with the pro-inflammatory cytokine TNFα, the miR-126 cargo of their extra-cellular vesicles was reduced by 80%, consistent with vesicular miR-126 reporting endothelial inflammation (Alexy et al., 2014). Reduced levels of circulating miR-126 have been described as a potential biomarker for vascular disorders such as diabetes (Jansen et al., 2013; Zampetaki et al., 2010) and myocardial infarction (Zampetaki et al., 2012). In patients with CKD, miR-126 has been reported to fall with worsening renal function (Fourdinier et al., 2019). miR-126 is also expressed in platelets (Kaudewitz et al., 2016) and its circulating level has been reported to reflect the circulating platelet count and activation state (Lorenzen et al., 2012; Willeit et al., 2013).

We hypothesized that circulating miR-126 would report vascular dysfunction in patients with kidney disease. As ‘proof-of-concept’, we first measured miR-126 in a relevant animal model, then in those with AAV. Finally, we measured miR-126 in patients with CKD and ESRD, as well as explored the relationship with established measures of endothelial function.

## Results

### Circulating miR in NTN model

**Figure 1:**
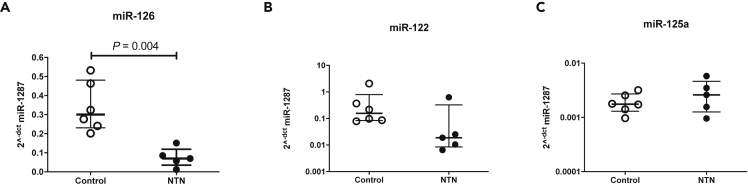
Circulating miRs in rats with nephrotoxic nephritis (NTN) and controls Each point represents the 2^−dct^ value (normalized by miR-1287) of circulating miR-126 (A), miR-122 (B) and miR-125a (C). The horizontal line represents the median and the bars represent the IQR. Significance determined by Mann-Whitney test.

In NTN rats, plasma miR-126 was reduced compared with untreated controls when the data were normalized by miR-1287 (Figure 1). This reduction in miR-126 was also reported by raw Ct values without any normalization (control Ct 27.6 [27.0–27.8], n = 6, NTN Ct 29.3 [28.4–30.1], n = 5, p = 0.02) There were no differences in miR-122 or miR-125a-5p between NTN and controls (Figure 1).

### Effect of blood processing

In healthy subjects, there was a significant difference in miR-126 concentration between plasma and serum. There was a significant decrease in miR-126 after blood storage for 24hr at both room temperature and at 4°C (Figure S1A). After 7d delay in processing, the concentration of miR-126 significantly increased relative to 24hr. Hemolysis accompanied this increase in miR-126 (for serum, no delay: A_414_ 0.22 [0.20–0.31]; 7d delay: A_414_ 1.80 [0.60–1.80], p = 0.03; for plasma, no delay: A_414_ 0.16 [0.16–0.28]; 7d delay: A_414_ 0.30 [0.22–0.45]; p = 0.03). Based on these data, only immediately processed plasma samples were used in all subsequent studies. After processing human blood into plasma or serum, storage at room temperature or 4°C for 24 hr or 7 days had no statistically significant effect on miR-126 concentration (Figure S1B). Therefore, after immediate isolation of plasma from blood, samples could be stored for short periods before analysis.

### Circulating miR in AAV

**Figure 2:**
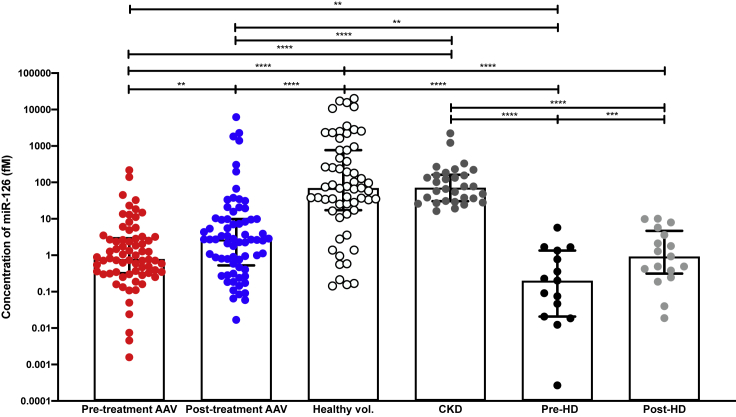
The concentration of miR-126 in patients with ANCA-associated vasculitis (AAV) (n = 70 paired) pre- and post-treatment, healthy volunteers (n = 60), chronic kidney disease (CKD) (n = 30) and end-stage renal disease (n = 15 paired) pre- and post-hemodialysis (HD) Bars show median with IQR, ∗∗p < 0.01, ∗∗∗∗p < 0.0001 (Mann-Whitney test for unpaired analysis and Wilcoxon test for paired analysis).

Seventy patients with AAV were recruited into this study. Pre- and post-treatment clinical data are shown in Table 1. No patients had diabetes or received anti-platelet treatment. Pre-treatment, the plasma miR-126 concentration was 88-fold lower than in healthy controls (AAV median 0.8 fM [IQR 0.3–2.9], healthy subjects 70.4 fM (17.2–770) (Figure 2). ROC analysis was performed to quantify the accuracy of miR-126 with regard to distinguishing health from AAV. The area under the curve was 0.87 (95% CI: 0.80–0.94). At a cutoff of >33fM, specificity was 96% (95% CI: 88–99) and sensitivity was 69% (57–80). Post-treatment, all 70 patients achieved disease remission (Table 1). Plasma miR-126 concentration increased 3.4-fold from pre-treatment levels (post-treatment median 2.7 fM [IQR 0.5–9.8]) but did not return to healthy levels. miR-122 plasma concentrations were not different between pre- and post-treatment samples from patients with AAV (pre-treatment 1.6 fM [0.7–3.4], post-treatment 1.8 fM [0.7–4.7]). There was no correlation between miR-126 and the patient's platelet count either pre- or post-treatment (Figure S2A).

**Table 1:** Clinical data for patients with AAV pre- and post-treatment

Characteristics	Pre-treatment (*n* = 70)	Post-treatment (*n* = 70)	p value
Age, years	62 ± 14 (27–82)	–	–
Sex, M/F	43/27	–	–
Organs involved	2 ± 1 (1–6)	–	–
Kidney	44	–	–
Lung	33	–	–
ENT	17	–	–
Nerve	13	–	–
Eyes	9	–	–
eGFR, mL/min/1.73m^2^	46 ± 34 (4–124)	57 ± 29 (9–121)	0.04
Creatinine, μmol/L	200 ± 177 (54–962)	150 ± 144 (64–1000)	0.03
ALT, U/L	20 ± 15 (6–83)	20 ± 10 (4–48)	0.94
ALP, U/L	96 ± 62 (13–338)	67 ± 18 (39–122)	<0.001
GGT, U/L	55 ± 44 (11–182)	33 ± 26 (10–148)	<0.001
Albumin, g/L	28 ± 7 (14–41)	37 ± 4 (24–46)	<0.0001
CRP, mg/L	82 ± 75 (3–275)	6 ± 15 (0–108)	<0.0001
Hemoglobin, g/L	107 ± 23 (62–157)	124 ± 28 (2–170)	<0.0001
Urine protein:creatinine mg/mmol	141 ± 188 (0–948)	107 ± 146 (0–531)	0.82
			

The data are shown as mean ± SD with range. Significance of numerical data between groups was ascertained using a 2-tailed paired t test.

**Figure 3:**
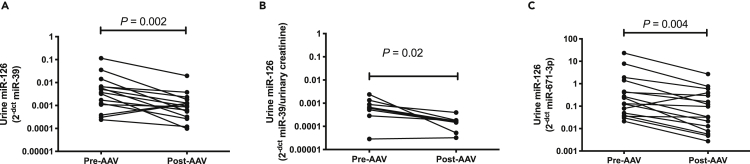
Urine miR-126 in patients with AAV, pre- (Pre-AAV) and post- (Post-AAV) treatment Each point represents the 2^−dct^ value (normalized by *C.elegans* miR-39 spike-in control (A), urinary creatinine concentration (B) and miR-671 (C)) of miR-126. Significance determined by Wilcoxon Test.

Urine miR-126 was measured pre- and post-immunosuppressive treatment (Figure 3). Using 3 different normalization methods (spike-in microRNA, urinary creatinine or internal microRNA) the urinary concentration of miR-126 consistently decreased post-treatment.

### Mechanism of miR-126 release into the circulation

**Figure 4:**
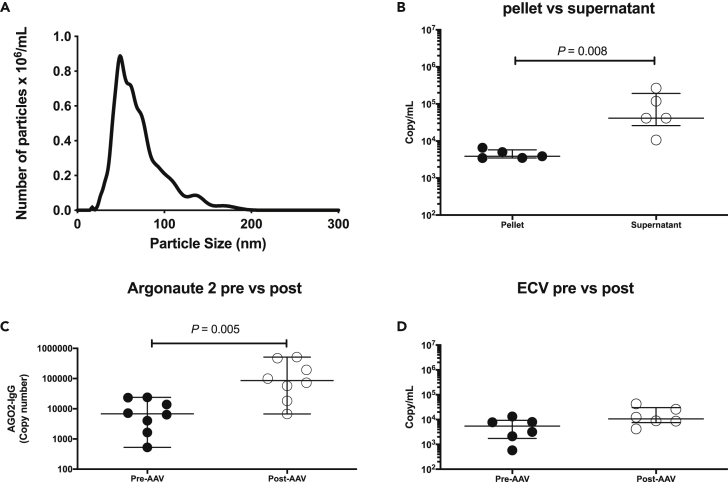
miR-126 is bound to argonaute 2 (Ago2) in human plasma (A) size and number of particles measured by nanoparticle tracking analysis (NTA) following isolation of extra-cellular vesicles from human plasma by differential centrifugation. (B) Dot plot of miR-126 measured in the extra-cellular vesicle containing pellet and supernatant after ultracentrifugation of human plasma. (C) Dot plot of miR-126 measured in the antibody isolated Ago2 fraction from 8 patients with AAV before treatment (Pre-AAV) and after treatment (Post-AAV). The y axis represents copy number obtained from the Ago2 pull-down minus the copy number obtained from IgG control pull-down from the same sample. (D) Dot plot of miR-126 measured in the extra-cellular vesicle containing pellet from 8 patients with AAV before treatment (Pre-AAV) and after treatment (Post-AAV). The horizontal line represents the median and the error bars represent the IQR. Significance determined by Wilcoxon Test.

miRs circulate bound to proteins or encapsulated in extra-cellular vesicles such as exosomes. Plasma from healthy volunteers was fractionated by differential centrifugation and extra-cellular vesicles were isolated (confirmed by nanoparticle tracking analysis – Figure 4A). miR-126 was enriched in the ‘protein fraction’ as opposed to the extra-cellular vesicle fraction (29-fold increase in supernatant compared to pellet) (Figure 4B). The RNA binding protein Ago2 was isolated from plasma and the bound miR-126 concentration measured. There was a significant increase in Ago2-bound miR-126 in those with AAV post-treatment (Figure 4C), consistent with this miR biomarker being specifically bound to Ago2. By contrast there was no significant treatment-induced change in miR-126 in the extra-cellular vesicle fraction (Figure 4D). There was no increase in Ago2-bound miR-122 and no change in plasma Ago2 concentration with treatment (data not shown).

### Circulating miR-126 in CKD

**Figure 5:**
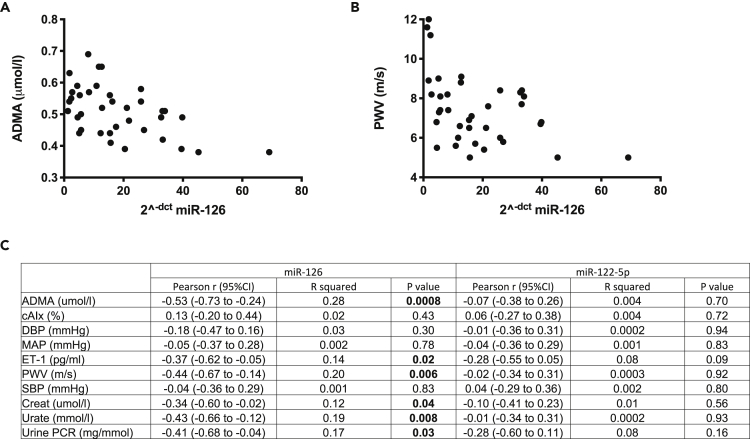
Scatterplots of asymmetric dimethylarginine (ADMA) (A and B) (A) and pulse wave velocity (PWV) (B) versus circulating miR-126 in patients with CKD. (C) Table with Pearson r (95% CI), r^2^ and p values of correlation between miR-126 and miR-122-5p and other biomarkers.

Having demonstrated proof-of-concept that miR-126 changes with acute endothelial/kidney injury, we explored its circulating concentration in CKD. This condition is associated with chronic endothelial dysfunction and high cardiovascular risk. There were no patients in this cohort with diabetes. Samples were analyzed from patients with CKD (n = 30) and hemodialysis-dependent ESRD (n = 15), immediately before and after dialysis (Table 2). In patients with ESRD, miR-126 was substantially reduced compared with healthy subjects and patients with CKD (352- and 358-fold, respectively). There was a modest increase following a hemodialysis session (4.7-fold) (Figure 2). There was a range of plasma miR concentrations in patients with CKD, which we hypothesized might reflect differences in vascular health across this patient group. For these patients, we correlated miR concentrations against a range of well-recognized measures of vascular function. miR-126 correlated significantly with PWV (r^2^ = 0.2), circulating ADMA (r^2^ = 0.28), ET-1 (r^2^ = 0.14) and urate concentrations (r^2^ = 0.19), as well as proteinuria (r^2^ = 0.17) (Figure 5). miR-126 correlated weakly with serum creatinine (r^2^ = 0.12). There was no relationship between miR-122 and any of the measures of vascular or renal function assessed. There was no correlation between miR-126 and the patient's platelet count either in the CKD or ESRD groups (Figures S2B and S2C).

**Table 2:** Clinical data obtained for CKD patients and ESRD patients pre-hemodialysis (HD)

Characteristics	CKD (*n* = 30)	Pre-HD (*n* = 15)
Age, years	56 ± 16 (26–82)	59 ± 12 (34–82)
Sex, M/F	21/9	8/7
eGFR, mL/min/1.73m^2^	25 ± 14 (7–57)	–
Creatinine, μmol/L	217 ± 133 (76–654)	–
ALT, U/L	19 ± 8 (9–50)	15 ± 6 (7–25)
ALP, U/L	97 ± 38 (40–182)	100 ± 43 (52–178)
CRP, mg/L	5 ± 2 (2–7)	14 ± 21 (2–77)
Hemoglobin, g/L	123 ± 16 (83–160)	122 ± 12.51 (94–139)

The data are shown as mean ± SD with range.

## Discussion

The current study develops the potential role of miR-126 as a circulating biomarker of vascular function in acute and CKD. We initially achieved proof of concept in rodents and then went on to study AAV as a prototype of severe, acute kidney injury and vascular dysfunction that is aggressively treated with immunosuppression. Then, we went on to study patients with CKD as they have a heavy burden of vascular dysfunction. In a clinically relevant rat model, a lower circulating miR-126 (but not other miR species) was associated with disease. We have demonstrated that in patients with AAV, circulating miR-126 concentrations are low at disease presentation and rise with successful treatment. Pre-treatment levels are lower than those seen in health and in CKD. Following treatment, miR-126 rises but not to levels seen in health. A liver-specific miR species (miR-122) showed no differences in patients with AAV before or after treatment. In patients with CKD, miR-126 correlated with other markers of vascular health. miR-126 was substantially reduced in patients with ESRD. Thus, miR-126 may be a useful marker of vascular inflammation and, with development, may help clinical decision-making.

miRs are an area of substantial research interest, in part because they represent potential disease biomarkers. It is widely believed that one of their key properties is that they are stable in the circulation (Mitchell et al., 2008). In the current study, we have demonstrated that for miR-126 this is not the case: significant degradation occurred if the sample was left unprocessed for 24hr. We have reported similar instability for miR-122 (Lopez-Longarela et al., 2020). We also demonstrated that hemolysis may increase the concentration of miR-126 present in the sample. Furthermore, despite reports that miRs are detected at similar concentrations in plasma and serum, plasma miR-126 was substantially higher than serum. Therefore, we recommend the immediate processing of plasma samples in future studies of miR-126.

miR-126 increased in the circulation following successful treatment of patients with AAV. There was no change in miR-122, a liver-specific miR not expressed in the endothelium. This is consistent with a specific change in the endothelium but deeper analysis of multiple miRs is required. In spot urine samples, miR-126 was reduced by treatment, which may contribute to the increase in the circulation. miR circulate encapsulated in extra-cellular vesicles such as exosomes and bound to proteins especially Ago2, but the relative contribution and biological importance of each fraction is controversial (Chevillet et al., 2014). In our study, when plasma was fractionated by centrifugation miR-126 predominately remained in the supernatant. This is consistent with studies that report a low amount of miR-126 in human exosomes (Chevillet et al., 2014). Importantly, we demonstrate here that miR-126 is bound to the circulating protein Ago2 and this fraction increases in patients with AAV following successful disease treatment. It is reported that stimulation of endothelial cells with TNFα resulted in a decrease of miR-126 (Alexy et al., 2014). This is in keeping with our own findings and suggests that the reduction in circulating miR-126 in those with active AAV may reflect a degree of endothelial dysfunction secondary to inflammation. This hypothesis is further supported by miR-126 being enriched in the endothelium (Harris et al., 2008) and our strong correlation between the circulating miR-126 and markers of endothelial function such as pulse wave velocity and ADMA. From a clinical perspective, a circulating biomarker that reports vascular health could have widespread utility and prospective studies should qualify miR-126 in a range of settings, including other systemic inflammatory disorders such as rheumatoid arthritis and systemic lupus erythematosus.

In keeping with our hypothesis that miR-126 might act as a measure of vascular integrity, circulating levels did correlate with well-recognized measures of endothelial dysfunction in our CKD cohort namely, high PWV, ADMA, ET-1, and urate. Plasma ADMA, an endogenous inhibitor of nitric oxide synthase, and plasma ET-1 were measured as components of the nitric oxide and ET systems, respectively. Both contribute to vascular dysfunction in CKD and an imbalance (more ET-1/less nitric oxide) may contribute to vasoconstriction, inflammation and atherosclerosis (Boger, 2003; Dhaun et al., 2006). Serum urate has also emerged as an important risk factor for cardiovascular risk and CKD progression (Feig et al., 2008). Treatment of asymptomatic hyperuricemia has been shown to improve renal function (Kanbay et al., 2007) and delay disease progression (Siu et al., 2006) in patients with early CKD. From a clinical perspective, ET receptor antagonism is being investigated as a novel therapeutic strategy for renoprotection in CKD (Dhaun and Webb, 2019). It has been shown to not only lower proteinuria but also serum urate (Dhaun et al., 2013; Heerspink et al., 2019). Furthermore, given there is often reciprocal up-regulation of the nitric oxide system when the ET system is down-regulated (Dhaun et al., 2006) an ET blocking strategy may offset some of the potentially deleterious effects of elevated circulating ADMA.

Interestingly, our data suggest no substantial difference in circulating miR-126 between health and CKD. The lack of a clear difference may relate to the CKD population we studied as they had minimal comorbidity without overt CVD. Additionally, we excluded those with diabetes. Indeed, the “vascular health” of our CKD subjects is demonstrated by a mean pulse wave velocity of 6.8 m/s, significantly lower than that of 8.2 m/s for a group with a similar eGFR reported in a study by Wang et al. (Wang et al., 2005). They used a similar technique for measuring arterial stiffness but included patients with diabetes and CVD. Furthermore, our subjects were relatively young (∼50 years), with good blood pressure control (∼135/80 mmHg) and reasonably preserved renal function (eGFR ∼75 mL/min). Thus, future studies should extend to the wider spectrum of CKD.

With further clinical development, miR-126 may have utility as a biomarker of vascular dysfunction in patients with kidney disease (and possibly in people with normal kidney function). Such a miR biomarker may provide a readout of cardiovascular drug efficacy that translates from pre-clinical models into early phase clinical trials. In support of this, we have demonstrated that miR-126 tracks effective treatment in patients with AAV and reports injury when translated back into a rat model of nephrotoxic nephritis. The work presented here provides proof of concept data to build on in larger studies that test utility, both in the specific clinical scenario of reporting disease activity in vasculitis, and across a broader group of patients with acute kidney injury and CKD.

### Limitations of the study

miR-126 is expressed in platelets and this represents an alternative source of miR-126 in the circulation. There was no correlation with the platelet counts across patient groups, but this does not exclude a difference in platelet activation state. While this cannot be excluded when comparing the AAV patients pre- and post-treatment it is unlikely to be a factor in the CKD group, where miR-126 correlated significantly with markers of endothelial dysfunction but not with platelet counts. Future studies could replicate our observations in platelet poor plasma as described by Sunderland et al. (Sunderland et al., 2017). We represent miR-126 in AAV as an absolute concentration generated from a standard curve using synthetic miR-126. The values generated allow comparison across groups, but the absolute value should be interpreted with caution as the reverse transcription efficiency may differ between synthetic target in buffer and plasma. Furthermore, release of miR-126 from platelets during sample processing could lead to higher apparent concentrations than are present in the cell-free fraction *in vivo.* Our data demonstrate that the Ago2 fraction of miR-126 changes with treatment of patients with AAV. To thoroughly characterize the role of extracellular vesicles, future studies should follow the guidelines published by the International Society of Extracellular Vesicles (Thery et al., 2018).

### Resource availability

#### Lead contact

Professor James Dear.

#### Materials availability

No new reagents were generated in this study.

#### Data and code availability

Data are available on request from the corresponding author.

## Methods

All methods can be found in the accompanying Transparent methods supplemental file.

## Supplemental information

Document S1. Transparent methods and Figures S1–S3
