# Supplementary material for: Circulating argonaute-bound microRNA-126 reports vascular dysfunction and treatment response in acute and chronic kidney disease
Source: iScience. 2020 Dec 13;24(1):101937. doi: 10.1016/j.isci.2020.101937 (PMC7773582; doi:10.1016/j.isci.2020.101937)
Supplement: Document S1. Transparent methods and Figures S1–S3 [file mmc1.pdf]

iScience, Volume *22*

Supplemental Information

Circulating argonaute-bound microRNA-126 reports vascular dysfunction and treatment response in acute and chronic kidney disease

Kathleen M. Scullion, A. D. Bastiaan Vliegenthart, Laura Rivoli, Wilna Oosthuyzen, Tariq E. Farrah, Alicja Czopek, David J. Webb, Robert W. Hunter, Matthew A. Bailey, Neeraj Dhaun, and James W. Dear

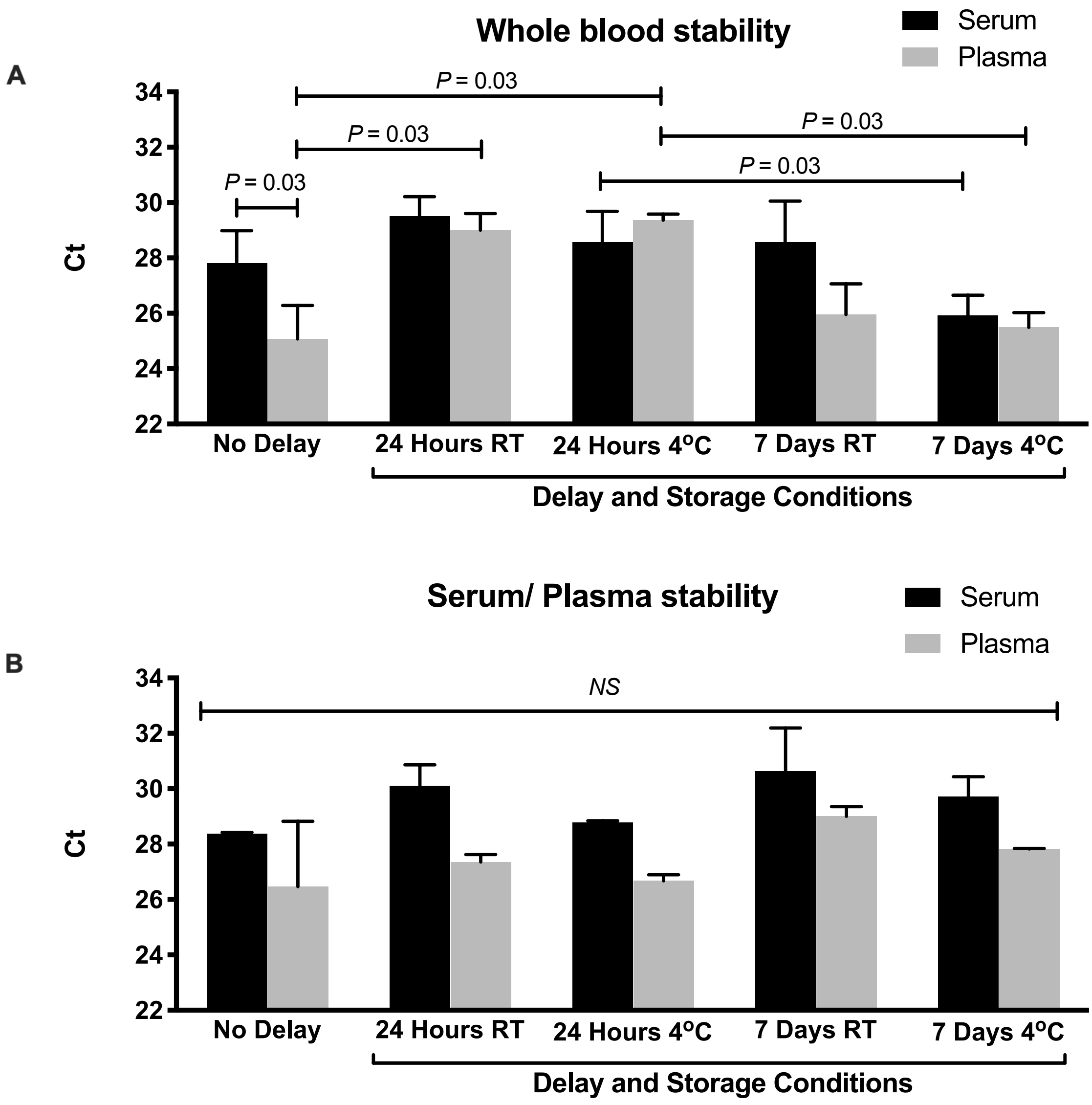

Supplementary figure 1: Effect of storage on serum & plasma miR-126. Related to figure 2.

A) Bar graphs displaying median Ct values of miR-126 measured by qPCR. Blood samples were collected from healthy volunteers (n=6). One serum and plasma blood tube was centrifuged and the supernatant frozen at -80 ^{0}C without delay. The remaining blood tubes were left at room temperature (RT) or 4^{0}C for 24h or 7 days before being processed and stored at -80 ^{0}C. B) Serum and plasma samples were immediately centrifuged to isolate serum/plasma. One serum and one plasma sample were stored at -80^{0}C without delay and the remaining tubes were left at room temperature (RT) or 4^{0}C for 24h or 7 days. Error bars represent the interquartile range.

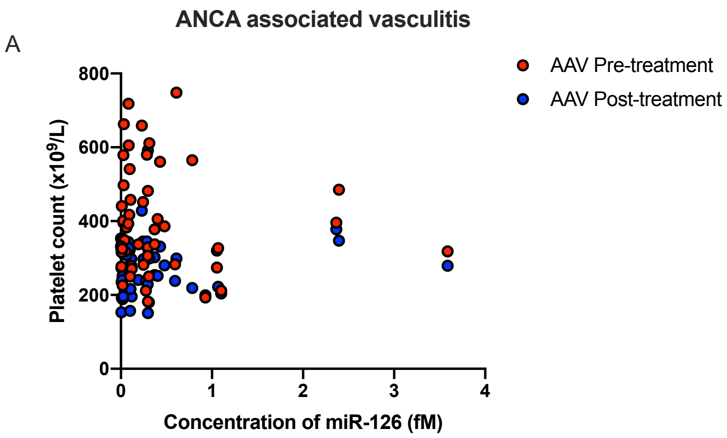

Pre-treatment Pearson r (95%CI) -0.07 (-0.33 to 0.20) P=0.6
Post-treatment Pearson r (95%CI) 0.09 (-0.19 to 0.36) P=0.5

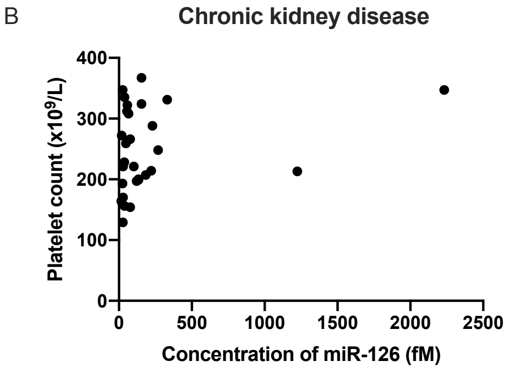

Pearson r (95%CI) 0.24 (-0.53 to 0.49) P=0.2

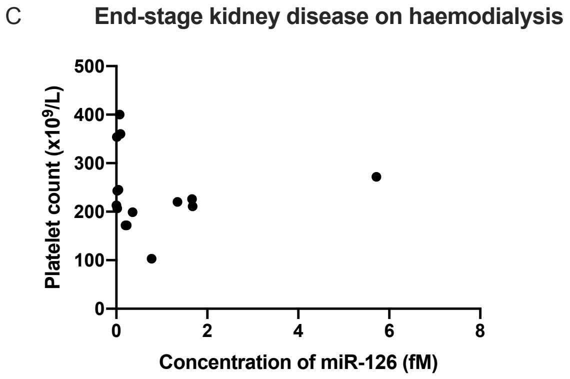

Pearson r (95%CI) -0.02 (-0.53 to 0.49) P=0.9

Supplementary figure 2: The relationship between plasma miR-126 and platelet count in patients with ANCA-associated vasculitis (AAV). Related to figure 2.

Pre and post treatment (A), chronic kidney disease (B) and End-stage kidney disease (C).

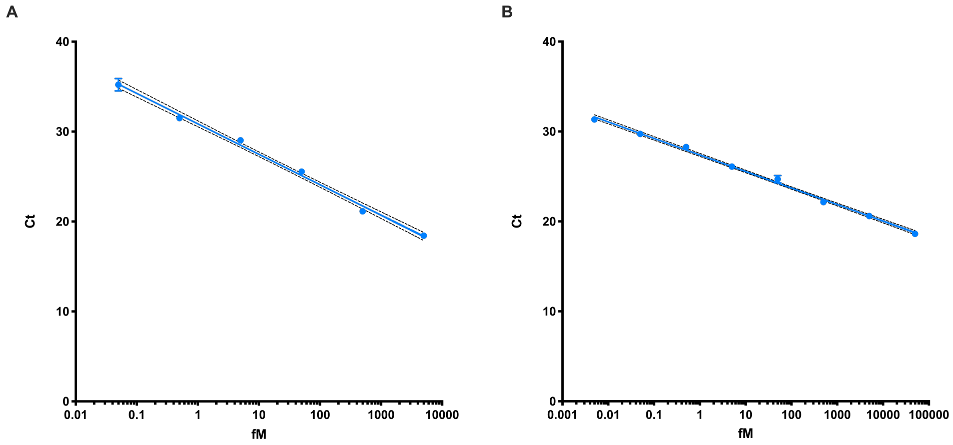

Supplementary figure 3: Standard curves used for absolute quantification of miRs. Related to Figure 2.

(A) miR-122 and (B) miR-126 standard curves for absolute quantification in patient samples. The solid blue line shows the semilog and the dotted line shows the 95% confidence interval (CI).

Transparent Methods

Participant groups

All studies were approved by the local research ethics committee and performed in accordance with the Declaration of Helsinki. The study, entitled “MicroRNA signatures of disease activity in ANCA-associated vasculitis”, REC reference 13/ES/0126 (formerly 10/S1402/33) was approved by The Tissue Governance committee in June 2015. Informed consent was obtained from all study participants.

Patients with ANCA Associated Vasculitis (AAV)

Patients presenting with AAV were recruited at the Royal Infirmary of Edinburgh, UK. Inclusion criteria were seropositivity for ANCA and organ-threatening disease requiring immunosuppression. Disease activity was graded according to the BVAS (scores range from 0 to 63, with higher scores indicating more active disease) (Mukhtyar et al., 2009) and by investigators’ assessments of disease activity as remission, ongoing active disease (treatment failure), or relapse. Remission was defined as a BVAS score of 0 that was maintained for 2 months and a prednisone dosage of ≤10 mg/day.

Patients with CKD

Subjects were recruited from the renal outpatient clinic at the Royal Infirmary of Edinburgh. The inclusion criteria were: male or female CKD patients, 18-65 years old and clinic BP ≤160/100 mmHg, whether or not on anti-hypertensive medication. We excluded patients with a renal transplant or on dialysis, patients with systemic vasculitis or connective tissue disease, those with a history of established cardiovascular disease, peripheral vascular disease, diabetes mellitus, respiratory disease, neurological disease, current alcohol abuse or pregnancy.

Patients with ESRD

Inclusion criteria were: age 18 or over, treated with haemodialysis (HD) for over 3 months. Patients affected by liver disease or with a history of hepato-biliary surgery were excluded; other exclusion criteria were consumption of cytochrome P450-inducing medications, past medical history of epilepsy, cancer, alcoholism and/or psychiatric disease. All patients were treated with HD for 4-5 hours per session, 3 times per week. Data collected included demographic characteristics, cause of ESRD, time on dialysis and current medications. As heparin in blood samples can inhibit polymerase chain reaction (PCR), blood samples were only used when the patient had not been exposed to heparin in the preceding 24 hours.

Healthy subjects

Adults with no medical complaints and no medication use were recruited and blood was drawn with informed consent.

Blood samples

In healthy subjects, blood was collected into 3 EDTA plasma (2.7 ml) and 3 serum (4.9 ml) tubes. One of each type of blood tube was processed without delay – by centrifugation at 1200 x *g* for 10 min at 4^0C and then supernatant then frozen at -80^0C. The remaining tubes of blood were left unprocessed at room temperature or 4^0C for 24h or 7 days. Hemolysis was quantified by spectrophotometric methods as described previously (Kirschner et al., 2013). In a second study plasma and serum was left at room temperature or 4^0C for 24h or 7 days.

In patients with AAV, blood samples were taken at study entry (before treatment) and at disease remission (as defined above). For CKD patients, samples were taken from a previously published study and were collected on the study day (Lilitkarntakul et al., 2011). Blood was collected into EDTA tubes and processed immediately as above. Urine was also collected and immediately frozen at -80^0C.

Plasma asymmetric dimethylarginine (ADMA), an endogenous inhibitor of nitric oxide synthesis, was measured using an optimised, fully validated high performance liquid chromatography method (intra- and inter-assay variations of 1.9% and 2.3%, respectively) (Blackwell et al., 2007). Plasma endothelin-1 (ET-1), the most potent endogenous vasoconstrictor, which contributes to CKD development and progression,(Dhaun et al., 2006) was determined by radioimmunoassay (Peninsular Laboratories Europe, St. Helens, UK) (assay variations 6.3% and 7.2%) (Strachan et al., 1999). The urate assay was based on the methods of Trivedi and Kabasakalian (Trivedi et al., 1978). Uric acid is oxidized to allantoin by uricase with the production of hydrogen peroxide (H_2O_2). The H_2O_2 reacts with 4-aminoantipyrine (4-AAP) and 2,4,6-tribromo-3-hydroxybenzoic acid (TBHB) in the presence of peroxidase to yield a quinoneimine dye. The resulting change in absorbance at 548 nm is proportional to the uric acid concentration in the sample. The limits of detection and quantification for the urate assay are 0.01 mmol/l and 0.015 mmol/l, respectively.

Extra-cellular vesicle isolation

Human plasma was fractionated by differential centrifugation to concentrate extra-cellular vesicles, as previously described (Chevillet et al., 2014). Plasma (1 mL) was centrifuged at 500 x *g* for 30 min then 12,000 x *g* for 20 min. The supernatant was then ultracentrifuged at 100,000 x *g* for 1h to pellet extra-cellular vesicles. The remaining supernatant is referred to as the ‘protein fraction’. The vesicles were re-suspended and then pelleted a second time by ultracentrifugation. Extra-cellular vesicle presence and number was quantified by nanoparticle tracking analysis as previously described (Oosthuyzen et al., 2013). miR concentration in each fraction was determined by PCR, described below.

Ago2 isolation

MagnaBind goat anti-mouse IgG magnetic bead slurry, 100 µL, (Thermo Scientific, Waltham, USA) was incubated with 10 µg of mouse monoclonal anti-Ago2 (Abcam, Cambridge, UK) or mouse normal IgG (Santa Cruz Biotechnology, Dallas, US) antibodies for 2h at 4°C. The

antibody-coated beads were then added to plasma and incubated overnight at 4°C with rotation. Beads were washed and each sample then eluted in RNAse free water before QIAzol was added for RNA isolation. Ago2 isolation was determined by Western blot analysis as described (Dear et al., 2011).

Measurement of arterial stiffness

In a subset of patients gold standard pulse wave velocity (PWV) was measured by the foot-to-foot wave velocity method using the SphygmoCor^{TM} system (SphygmoCor^{TM} Mx, AtCor Medical, Sydney, Australia, version 6.31), in which a high-fidelity micromanometer (SPC-301, Millar Instruments, Texas, USA) was used to determine carotid-femoral PWV (Oliver and Webb, 2003).

Measurement of plasma and urine miR

The following miR were measured: miR-126-3p, miR-122-5p, miR-1287 and miR-671.

RNA extraction

RNA was extracted from each sample (50 µl) using the miRNeasy serum/plasma kit (Qiagen, Venlo, Netherlands).

PCR

After RNA extraction, 5 µl of each eluate was reverse transcribed into cDNA using the miScript II RT Kit (Qiagen, Venlo, Netherlands). The synthesised cDNA was ten-fold diluted and used for cDNA template in combination with the miScript SYBR Green PCR Kit (Qiagen, Venlo, Netherlands) using the specific miScript assays (Qiagen, Venlo, Netherlands). Real-time PCR was performed on a Light Cycler 480 (Roche, Basel, Switzerland) using the recommended miScript cycling parameters.

Absolute miRNA quantification

Absolute quantification of miRs was achieved by generating a standard curve using synthetic target. Standard curves were generated by reverse transcribing known concentrations of miScript miRNA mimics (Qiagen, Venlo, The Netherlands) in 0.1X TE buffer spiked with 10 ng/µl Poly-C (Sigma-Aldrich, Gillingham, UK). The resulting cDNA was measured using serial dilutions on 3 different plates on three different days to demonstrate minimal variability (Supplementary Figure 3).

Relative quantification

MiR expression was analysed using the DCt method (Schmittgen and Livak, 2008). A *C. elegans* miR mimic was used as a spiked-in control (miR-39). This allowed for the Ct values for the miRs of interest to be normalised to the spiked-in control. The data obtained were translated by the 2^{-DCt} method.

Statistical analysis

Data are presented as mean ± standard deviation for patient characteristics and as median and interquartile range (IQR) for all other datasets. Each dataset was analysed for normality using a Shapiro-Wilk test. For non-parametric datasets, comparisons were made using the Mann-Whitney test or Wilcoxon matched-pairs signed rank test. For the study of patients with ANCA vasculitis, with 70 patients the probability is 80% that the study will detect a pre- and post-treatment difference in miR-126 of 65 at a two-sided 0.05 significance level. This is based our pilot data that demonstrated a standard deviation of the change in miR-126 pre- and post-treatment of 190. In our pilot data the mean change in miR-126 was 125 and a change of 65 was deemed an acceptable level of detection.

Rat model of nephrotoxic nephritis (NTN)

NTN was induced by raising a nephrotoxic serum (NTS) in rabbits to isolated sonicated rat glomeruli which was then injected into male Sprague-Dawley rats (1mL/200 g). A telescoped model of NTN was used where the rats are pre-immunised with 1 mg rabbit immunoglobulin before the injection of NTS (Holdsworth et al., 1981).

Supplemental References

Blackwell, S., O'Reilly, D.S., and Talwar, D. (2007). Biological variation of asymmetric dimethylarginine and related arginine metabolites and analytical performance goals for their measurement in human plasma. European Journal of Clinical Investigation *37*, 364-371.

Chevillet, J.R., Kang, Q., Ruf, I.K., Briggs, H.A., Vojtech, L.N., Hughes, S.M., Cheng, H.H., Arroyo, J.D., Meredith, E.K., Gallichotte, E.N.*, et al.* (2014). Quantitative and stoichiometric analysis of the microRNA content of exosomes. Proceedings of the National Academy of Sciences of the United States of America *111*, 14888-14893.

Dear, J.W., Simpson, K.J., Nicolai, M.P., Catterson, J.H., Street, J., Huizinga, T., Craig, D.G., Dhaliwal, K., Webb, S., Bateman, D.N.*, et al.* (2011). Cyclophilin A is a damage-associated molecular pattern molecule that mediates acetaminophen-induced liver injury. J Immunol *187*, 3347-3352.

Dhaun, N., Goddard, J., and Webb, D.J. (2006). The endothelin system and its antagonism in chronic kidney disease. Journal of the American Society of Nephrology *17*, 943-955.

Holdsworth, S.R., Neale, T.J., and Wilson, C.B. (1981). Abrogation of macrophage-dependent injury in experimental glomerulonephritis in the rabbit. Use of an antimacrophage serum. The Journal of Clinical Investigation *68*, 686-698.

Kirschner, M.B., Edelman, J.J., Kao, S.C., Vallely, M.P., van Zandwijk, N., and Reid, G. (2013). The Impact of Hemolysis on Cell-Free microRNA Biomarkers. Frontiers in Genetics *4*, 94.

Lilitkarntakul, P., Dhaun, N., Melville, V., Blackwell, S., Talwar, D.K., Liebman, B., Asai, T., Pollock, J., Goddard, J., and Webb, D.J. (2011). Blood pressure and not uraemia is the major determinant of arterial stiffness and endothelial dysfunction in patients with chronic kidney disease and minimal co-morbidity. Atherosclerosis *216*, 217-225.

Mukhtyar, C., Guillevin, L., Cid, M.C., Dasgupta, B., de Groot, K., Gross, W., Hauser, T., Hellmich, B., Jayne, D., Kallenberg, C.G.*, et al.* (2009). EULAR recommendations for the management of primary small and medium vessel vasculitis. Annals of the Rheumatic Diseases *68*, 310-317.

Oliver, J.J., and Webb, D.J. (2003). Noninvasive assessment of arterial stiffness and risk of atherosclerotic events. Arteriosclerosis, thrombosis, and vascular biology *23*, 554-566.

Oosthuyzen, W., Sime, N.E., Ivy, J.R., Turtle, E.J., Street, J.M., Pound, J., Bath, L.E., Webb, D.J., Gregory, C.D., Bailey, M.A.*, et al.* (2013). Quantification of human urinary exosomes by nanoparticle tracking analysis. The Journal of Physiology *591*, 5833-5842.

Schmittgen, T.D., and Livak, K.J. (2008). Analyzing real-time PCR data by the comparative C(T) method. Nat Protoc *3*, 1101-1108.

Strachan, F.E., Spratt, J.C., Wilkinson, I.B., Johnston, N.R., Gray, G.A., and Webb, D.J. (1999). Systemic blockade of the endothelin-B receptor increases peripheral vascular resistance in healthy men. Hypertension *33*, 581-585.

Trivedi, R.C., Rebar, L., Berta, E., and Stong, L. (1978). New enzymatic method for serum uric acid at 500 nm. Clinical Chemistry *24*, 1908-1911.
